# Evaluation of a Patient-Collected Audio Audit and Feedback Quality Improvement Program on Clinician Attention to Patient Life Context and Health Care Costs in the Veterans Affairs Health Care System

**DOI:** 10.1001/jamanetworkopen.2020.9644

**Published:** 2020-07-31

**Authors:** Saul Weiner, Alan Schwartz, Lisa Altman, Sherry Ball, Brian Bartle, Amy Binns-Calvey, Carolyn Chan, Corinna Falck-Ytter, Meghana Frenchman, Bryan Gee, Jeffrey L. Jackson, Neil Jordan, Benjamin Kass, Brendan Kelly, Nasia Safdar, Cecilia Scholcoff, Gunjan Sharma, Frances Weaver, Maria Wopat

**Affiliations:** 1 Department of Medicine, University of Illinois at Chicago, Chicago; 2 Center of Innovation for Complex Chronic Healthcare, Jesse Brown VA Chicago Health Care System, Chicago, Illinois; 3 Department of Medical Education, University of Illinois at Chicago, Chicago; 4 Office of Healthcare Transformation, VA Greater Los Angeles Healthcare System, Los Angeles, California; 5 Research Services, Louis Stokes Cleveland VA Medical Center, Cleveland, Ohio; 6 Center of Innovation for Complex Chronic Healthcare, Edward Hines Jr VA Hospital, Hines, Illinois; 7 Department of Medicine, Yale University School of Medicine, New Haven, Connecticut; 8 Primary Care, Louis Stokes Cleveland VA Medical Center, Cleveland, Ohio; 9 Department of Medicine, VA Greater Los Angeles Healthcare System, Los Angeles, California; 10 Department of Medicine, Edward Hines Jr VA Hospital, Hines, Illinois; 11 General Medicine Division, Clement J. Zablocki VA Medical Center, Milwaukee, Wisconsin; 12 Department of Medicine, Medical College of Wisconsin, Milwaukee, Wisconsin; 13 Department of Psychiatry and Behavioral Sciences and Preventive Medicine, Northwestern University Feinberg School of Medicine, Chicago, Illinois; 14 Research Services, William S. Middleton Memorial Veterans Hospital, Madison, Wisconsin; 15 Department of Public Health Sciences, Loyola University Chicago, Chicago, Illinois; 16 Pharmacy Services, William S. Middleton Memorial Veterans Hospital, Madison, Wisconsin

## Abstract

**Importance:**

Evidence-based care plans can fail when they do not consider relevant patient life circumstances, termed *contextual factors*, such as a loss of social support or financial hardship. Preventing these contextual errors can reduce obstacles to effective care.

**Objective:**

To evaluate the effectiveness of a quality improvement program in which clinicians receive ongoing feedback on their attention to patient contextual factors.

**Design, Setting, and Participants:**

In this quality improvement study, patients at 6 Department of Veterans Affairs outpatient facilities audio recorded their primary care visits from May 2017 to May 2019. Encounters were analyzed using the Content Coding for Contextualization of Care (4C) method. A feedback intervention based on the 4C coded analysis was introduced using a stepped wedge design. In the 4C coding schema, clues that patients are struggling with contextual factors are termed *contextual red flags* (eg, sudden loss of control of a chronic condition), and a positive outcome is prospectively defined for each encounter as a quantifiable improvement of the contextual red flag. Data analysis was performed from May to October 2019.

**Interventions:**

Clinicians received feedback at 2 intensity levels on their attention to patient contextual factors and on predefined patient outcomes at 4 to 6 months.

**Main Outcomes and Measures:**

Contextual error rates, patient outcomes, and hospitalization rates and costs were measured.

**Results:**

The patients (mean age, 62.0 years; 92% male) recorded 4496 encounters with 666 clinicians. At baseline, clinicians addressed 413 of 618 contextual factors in their care plans (67%). After either standard or enhanced feedback, they addressed 1707 of 2367 contextual factors (72%), a significant difference (odds ratio, 1.3; 95% CI, 1.1-1.6; *P* = .01). In a mixed-effects logistic regression model, contextualized care planning was associated with a greater likelihood of improved outcomes (adjusted odds ratio, 2.5; 95% CI, 1.5-4.1; *P* < .001). In a budget analysis, estimated savings from avoided hospitalizations were $25.2 million (95% CI, $23.9-$26.6 million), at a cost of $337 242 for the intervention.

**Conclusions and Relevance:**

These findings suggest that patient-collected audio recordings of the medical encounter with feedback may enhance clinician attention to contextual factors, improve outcomes, and reduce hospitalizations. In addition, the intervention is associated with substantial cost savings.

## Key Points

**Question:**

Can data from patient-collected audio recordings of their visits help clinicians improve their attention to patient life context when planning care, improve patient outcomes, and reduce health care costs?

**Findings:**

In this quality improvement study, feedback was delivered to 666 clinicians based on analysis of 4496 audio-recorded visits. Attention to patient contextual factors increased from 67% to 72%, and contextualized care planning was associated with a greater likelihood of improved outcomes, resulting in an estimated cost savings of $25.2 million from avoided hospitalizations.

**Meaning:**

These findings suggest that continuous feedback to clinicians about their attention to patient life context, based on audio recordings of their care, may substantially improve their performance, with measurable benefits for their patients and substantial cost savings.

## Teaser

This quality improvement study evaluates the effectiveness of a program in which clinicians at Veterans Affairs (VA) clinics receive ongoing feedback on their attention to patient contextual factors via patient-collected, audio-recorded sessions.

## Introduction

Contextualizing care is the process of adapting research evidence to patient context.^1,2^ For example, recognizing that a patient has lost control of their diabetes because they cannot afford insulin glargine and switching them to a less costly alternative is a contextualized care plan. Conversely, the failure to adapt research evidence to patient context, when it results in an inappropriate plan of care, is termed a *contextual error*.^3,4^ An attempt to improve diabetes control by increasing the dosage of medication that a patient cannot afford, and therefore has not taken as directed, would represent a contextual error.

Contextual errors occur frequently,^5,6^ adversely impact health care outcomes,^6^ and can increase health care costs.^7^ Nevertheless, they are difficult to detect because they are rarely discoverable from reviewing the medical record. In the aforementioned example, the physician is likely to document in their note that they responded to the patient’s poor diabetes control by increasing their insulin glargine dosage, which would seem reasonable. They are not likely to document that the patient cannot afford the medication, because they never elicited that information.

A more reliable way to ascertain whether a care plan was contextualized is to audio record the encounter, in addition to reviewing the medical record of that encounter. An efficient method for collecting audio recordings of encounters is to invite patients to carry audio recorders into their visits.^8^ Encounters are then analyzed using a coding method called Content Coding for Contextualization of Care (4C),^9^ which has demonstrated 85% interrater agreement for differentiating a contextualized care plan from a contextual error across trained coders. Coders follow a 4-step process of looking for clues that a patient is struggling (ie, *contextual red flags*) and then listening for whether the physician asked about them (ie, *contextual probes*), whether relevant life circumstances were identified (ie, *contextual factors*), and whether there was an attempt to address them in the care plan.

In a study^6^ of approximately 600 encounters using 4C analysis, contextual red flags were more likely to resolve or improve at 4 to 6 months after an audio-recorded visit if the care plan had been contextualized. Examples of such context-specific good outcomes, taken from the 4C coding manual,^10^ are shown in Table 1. The study provided observational data on the benefits of contextualizing care.

**Table 1.** Examples of Prospectively Defined Good vs Poor Outcomes for Contextual Red Flags Extracted From the Medical Record or Heard on the Audio Recording^a^

Red flags	Good outcome	Poor outcome
Medical record		
Diabetes: glycated hemoglobin A_1C_ >8	Any improvement in glycated hemoglobin A_1C_	No improvement or glycated hemoglobin A_1C_ is higher
Hypertension: systolic BP >140 mm Hg or diastolic BP >90 mm Hg	Any improvement in systolic BP or diastolic BP	No improvement or BP is higher
Missed appointments: ≥2 in past 4 mo	Patient makes it to next scheduled appointment	Patient misses next scheduled appointment
Missed medications: ≥1 missed fills or refills in past 4 mo	Patient’s medication is filled or refilled	Patient’s medication not filled or refilled
Missed laboratory tests or procedures: ≥1 or more missed in past 4 mo	Patient obtains laboratory tests or recommended procedures	Patient does not obtain laboratory tests or recommended procedures
Audio recording		
Medications: discovered during visit that patient has run out, stopped taking, or has expired medications	Patient is adherent with their medications	Patient is nonadherent with their medications
Understanding: patient indicates confusion about how to make appointments, get laboratory tests, get to clinic, and so forth	Patient completes appointments, laboratory tests, and so forth	Patient misses appointments, laboratory tests, and so forth
Discrepancies: patient reports different BP or blood glucose levels at home than at clinic	Patient reports BP or blood glucose levels consistent with readings at clinic	Patient continues to report different levels at home
Refusal: patient refuses colonoscopy or recommended vaccines (eg, influenza)	Patient obtains recommended vaccines or procedures	Patient does not get recommended vaccines or procedures

Abbreviation: BP, blood pressure.

^a^ Examples shown are from the Content Coding for Contextualization of Care Coding Manual by Weiner et al.^10^

**Figure 1.**
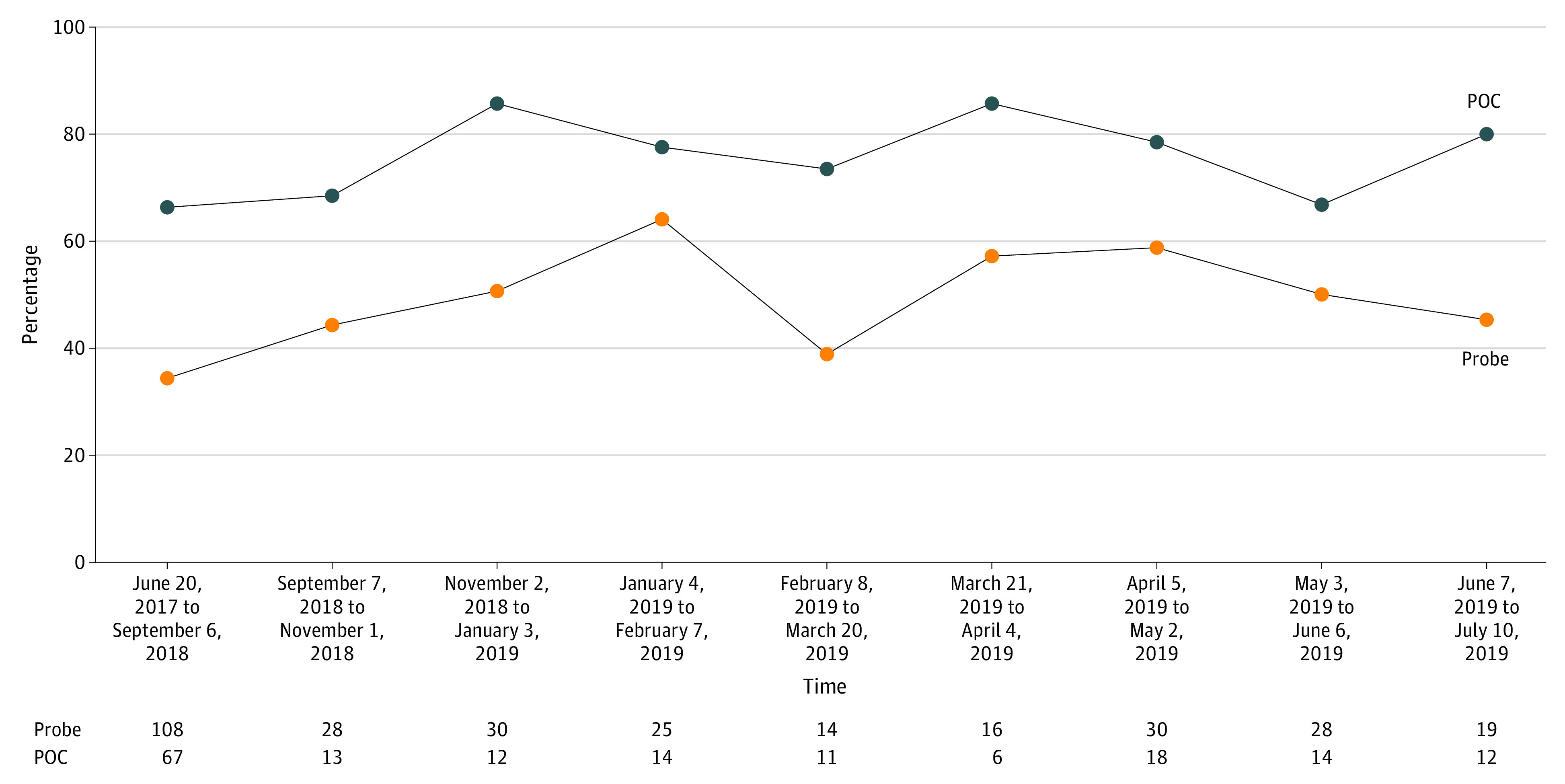
Sample Report Chart Example from a run chart tracking contextual probing (Probe) and contextualization of the plan of care (POC) at site E. Probe is the percentage of red flags heard by coders that were probed by the physician; POC is the percentage of contextual factors heard by coders that were incorporated into the plan of care by the physician.

On the basis of these data, in 2013 the Department of Veterans Affairs (VA) introduced a quality improvement (QI) program at 2 of the sites in this study in which physicians receive ongoing feedback on their performance at contextualizing care, tracking 2 metrics: the percentage of audio-recorded encounters in which contextual red flags were probed when present and the percentage of recorded encounters in which contextual factors were addressed in the care plan when present (Figure 1). Feedback to clinicians consisted of a 4-line summary of each recorded visit indicating the contextual red flag, the contextual probe (if any), the contextual factor (if any), and the contextualized or noncontextualized care plan (see examples in Table 2), and a run chart (Figure 1) showing the 2 tracked metrics trended over time. Feedback was offered at 2 levels of intensity, starting with standard feedback, consisting of monthly reports to physicians with anonymized case examples discussed at monthly meetings and a case-of-the-week emailed to all clinical staff, followed by the expansion of the program (enhanced feedback) to include nurses and clinical pharmacists in the same practice groups, residents when present, online reflective exercises for continuing medical education (CME) and board recertification credit based on the recorded cases, optional individualized reports, and inclusion of data on the outcomes of contextual red flags (Table 2).

**Table 2.** Examples of a Contextualized Care Plan and a Contextual Error

Contextualized care plan	Contextual error
1. Contextual red flag: a diabetic patient’s glycated hemoglobin A_1C_ had gone up (7.2 to 9.1)	1. Red flag: a diabetic patient’s glycated hemoglobin A_1C_ had gone up (6.8 to 8.3)
2. Contextual probe: the physician asked the patient why	2. No probe: the clinician did not ask why
3. Contextual factor: the patient explained that their medications were stolen twice from their apartment entryway	3. Contextual factor revealed by patient: the patient mentioned that he was no longer playing basketball because he had a knee injury
4. Contextual plan of care: the clinician rerouted medications to the facility pharmacy	4. Contextual plan of care: the clinician examined the knee and prescribed pain medication but did not discuss strategies for exercising without stressing the knee
Outcome of red flag: at 4 mo the patient’s glycated hemoglobin A_1C_ came down to 7.4	Outcome of red flag: at 5 mo, the patient’s glycosylated hemoglobin A_1C_ was up to 8.7

We report here a quality improvement study designed to address the following questions^11^: Does a patient-collected audio program with audit and feedback of data on contextualization of care prevent contextual errors? If so, is the reduced error rate associated with improved health care outcomes 4 to 6 months later as defined by resolution or partial resolution of contextual red flags identified at the audio-recorded visit (Table 1)? Finally, how do the costs of the program compare with cost savings that can be attributed to the program? For the latter, we focused on emergency department visits and hospitalization rates of patients whose physicians participated in the program. We selected these because they are dichotomous, unambiguous outcome measures that reflect the effectiveness of outpatient care for many conditions and substantially drive costs.

## Methods

This study was approved by the VA Central Institutional Review Board. The program was approved by the Quality Improvement Committee or comparable authority at each site. Patients who participated in the audio recordings provided written, informed consent. This study follows the Standards for Quality Improvement Reporting Excellence (SQUIRE) reporting guideline.

**Figure 2.**
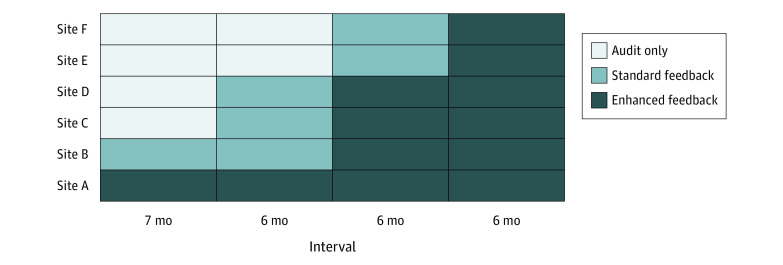
Stepped Wedge Design The term *audit* refers to the period during which baseline data was collected on contextualization of care rates (at sites C-F) with no feedback to clinicians.

Ambulatory care clinics based at 6 VA medical centers participated in this QI study (Figure 2). All were primary care clinics, except at site E, where a gastroenterology clinic was included. The program had been intermittently operational at 2 of the sites (sites A and B) since 2013, depending on leadership support and funding. At site A, the program was active at the start of the study, with clinicians receiving enhanced feedback. At site B, clinicians had received standard feedback before the start date, but the program had been inactive for the prior 26 months. Sites C, D, E, and F had no prior exposure to the program and were added sequentially, as illustrated in Figure 2. After a 7-month audit-only period for the 4 new sites, sites C and D began standard feedback, whereas sites E and F remained audit only. At 6-month intervals, paired sites advanced until all were receiving enhanced feedback (at site B, pharmacists and nurses did not participate). Assignment of sites C through F, each a cluster, to the steps was randomized.

In each participating clinic, a project assistant and trained volunteers staffed a table in the waiting room with flyers and posters advertising the program. Patients who expressed interest were informed that they could audio record their visit using an encrypted audio recorder for a program designed to help clinicians improve the quality of patient care. They could either carry it out in the open, or conceal it if they preferred (at site B, patients were instructed to reveal the recorder per site-specific protocol), and turn it off at any time. Participants in the QI program signed a consent form and returned the audio recorder after their visit as they exited the clinic. The recordings were uploaded to a secure server, and a team of 4C-trained coders with access to the patient medical record coded the encounter, identifying contextual red flags, contextual probes, contextual factors, and contextualized care plans, where present. To determine the outcome, 4 to 6 months after each encounter, a member of the coding team looked in the patient’s medical record for the status of any identified contextual red flags and noted whether they had improved, using predetermined criteria in the 4C coding manual^10^ (Table 1). To avoid bias, the coder was blinded as to whether the care plan for the encounter had been coded as contextualized.

Before the initiation of standard feedback, at least 1 clinical champion was designated at each site. The clinical champion was a peer—typically a primary care physician—who facilitated the discussion of data generated from the 4C coding. Clinical champions received training on giving feedback either during a 1-day workshop at the start of the study, or through coaching sessions over the telephone. Approximately once per month, each clinical champion received an electronic slide presentation emailed by the coding team, with each slide summarizing the 4 essential elements of an audio recording (Table 2). Slides were organized in a consistent format starting with examples in which the physician failed even to probe a contextual red flag to those in which they successfully performed all needed steps to arrive at a contextualized care plan. Champions presented the slides and facilitated discussion at standing clinical meetings at their facility. In addition, the coding team posted a weekly case on the clinician’s listserve, alternating between examples of contextual errors and effectively contextualized care plans.

When it was time to introduce enhanced feedback, audio coders would begin listening to and coding sections of audio recordings that included interactions with nurses and pharmacists and adding slides of those interactions to the slide presentations. They also included data on the outcome for each example at the bottom of each slide (Table 2). After the feedback sessions, attending physicians were emailed an optional worksheet with questions to address related to 1 or more cases discussed, indicating changes they would make to their practice. Participation was awarded with both CME credit and maintenance of certification credit from the American Board of Internal Medicine. Physicians who requested it were sent examples from their own clinics. Feedback to residents was provided either by the clinical champion or someone else designated by the residency program director or chief resident.

The program implementation process was guided by 3 principles: that it not be burdensome to clinicians or patients, that it feel safe to all participants, and that they see value in participating. Hence, feedback occurred only during pre-existing standing meetings, data were deidentified by the coding team before sharing, and physicians received CME and board recertification credits where applicable. As with most operationally approved QI projects, nearly all clinicians were enrolled, with the exception of site B, where the QI committee authorized an opt-in program only.

Research tasks conducted for the purpose of assessing the effectiveness of the QI program consisted of analysis of data generated by the 4C coding and a budget analysis. The latter was conducted by maintaining a log of all staff time and other resources for all activities related to the program at all participating sites and centrally (eg, the 4C coding) for the purpose of tabulating expenses. Prorated salaries of all staff, including project assistants who handed out and collected audio recorders and the coders, were calculated and tabulated. Data on emergency department and inpatient rates for patients of physicians participating in the QI program were extracted from the VA Corporate Data Warehouse, using codes linking patients to their clinician.

### Statistical Analysis

We fitted mixed-effects logistic regression models to estimate the likelihood of (1) probing each red flag, (2) addressing each revealed contextual factor, and (3) improvement in presenting red flags after 4 to 6 months. Models included the step (baseline vs any feedback, standard vs enhanced feedback) as the primary factor and controlled for time in the study, study site, and hierarchal clustering of red flags and factors within patients and patients within clinicians. Sample size calculations based on prior findings suggested that a total of 408 recordings with contextual factors (and additional recordings with red flags but not factors) would provide 80% power to detect past effect sizes (eg, a change from 45% to 65% likelihood); hence, we aimed to collect 2040 recordings (408 per site for the 5 sites that were in each group) to be able to examine hypotheses within each site.

We estimated cost savings by fitting Cox proportional hazard models to the time from outpatient visit to first emergency visit or hospitalization, with step as the key factor and covariates for site and patient sex, race, age group, and risk (via the Nosos Score) and multiplying the estimated annualized difference in hospitalizations among the clinicians’ panels by the mean site-specific cost of hospitalization in 2018 dollars.^12^ Adjusted hazard ratios in the Cox models were tested using 2-sided *z* tests based on robust SEs, with *P* < .05 as the significance threshold. Analyses were conducted in R statistical software version 3.6 (R Project for Statistical Computing) and Stata statistical software version 15.1 (StataCorp). Data analysis was performed from May to October 2019.

## Results

From May 1, 2017, to May 22, 2019, 4496 recordings were made by patients across the 6 participating sites during visits with 666 clinicians (Table 3). Patients seen in the participating clinics had a mean age of 62.0 years of age, 92% were male, 68% were White, 21% were non-Hispanic African-American, and 6% were Hispanic/Latino. A total of 2994 audio recordings (67%) contained at least 1 contextual red flag. Among these, 6860 red flags were identified, of which 3768 were probed (55%), identifying 2164 contextual factors (57%). An additional 821 contextual factors were revealed by patients without probing.

**Table 3.** Number of Audio Recordings by Site and Step

Site	Audio recordings, No.		
	Baseline	Standard	Enhanced
A	0	0	1187
B	100	291	320
C	303	200	376
D	86	70	323
E	306	177	286
F	287	80	104

Before analyzing the step effects, we determined whether the patient revealing the recorder during the visit was associated with clinician probing or planning using mixed-effects logistic regression models; there was no association with either probing (odds ratio [OR], 0.98; 95% CI, 0.87-1.11; *P* = .79) or planning (OR, 0.97; 95% CI, 0.80-1.19; *P* = .81). The likelihood of a clinician probing red flags increased with standard or enhanced feedback (adjusted OR, 1.5; 95% CI, 1.3-1.8; *P* < .001).

Before receiving feedback, clinicians addressed 413 of 618 contextual factors in their care plans (67%). After receiving either standard or enhanced feedback, they addressed 1707 of 2367 contextual factors (72%; OR, 1.3; 95% CI, 1.1-1.6; *P* = .01). When we controlled for probing, clinicians were more likely to contextualize the care plan in the enhanced than in the standard feedback condition (adjusted OR, 1.9; 95% CI, 1.4-2.7; *P* < .001).

At the medical record review 4 to 6 months after the visit, measured outcomes for contextual red flags were available for 1227 of the contextualized care plans, of which 901 (73%) were good, as defined by partial or full resolution of the contextual red flag (Table 1). Measured outcomes were available for 474 of the noncontextualized care plans, of which 218 (46%) were good. When we controlled for step and clustering in patients and clinicians, contextualized care planning was associated with improved outcomes (adjusted OR, 2.5; 95% CI, 1.5-4.1; *P* < .001).

In the budget analysis, the Cox regression models found no difference in emergency department visit rate among patients according to their clinicians’ exposure to feedback interventions, but found a significant decrease in the rate of hospitalization from 19.0% at baseline to 16.5% for patients seeing clinicians in the enhanced feedback step (adjusted hazard ratio, 0.93; 95% CI, 0.88-0.98; *P* = .03). As a result, we estimate that 987 hospitalizations were avoided across the 6 sites (approximately 2.5% of annual hospitalizations of patients of the clinicians), at a savings of approximately $25.2 million (95% CI, $23.9-$26.6 million) based on per-site hospitalizations avoided and per-site 2018 costs of an average hospitalization. The total cost of the intervention was $337 242.

## Discussion

Providing ongoing feedback to clinicians on their attention to patient life context based on data collected from audio recordings and the medical record may improve their performance at contextualizing care. Patient face challenges with refilling medications, making it to appointments, and understanding their treatment plans, among other things. When physicians see that their efforts to address these challenges are effective at resolving them, they may change how they practice. This change benefits patients by increasing the chances that contextual red flags (eg, not refilling medication or missing appointments) improve or resolve. Sites that participated in the enhanced feedback step had reduced hospitalization rates, with cost savings greater than the cost of the intervention.

It may not be surprising that when physicians take steps to address patient life challenges complicating care, those patients often benefit from the intervention. Contextualizing care may be regarded as the pragmatic effort of the clinician to address social determinants of health during the medical encounter.^13^ In their recent report^13^ on integrating social care on the delivery of health care, the National Academy of Medicine identified 5 complementary facilitating activities: awareness, adjustment, assistance, alignment, and advocacy. Two of them, awareness and assistance, correspond to identifying contextual red flags (eg, determining that a patient is missing appointments because of a transportation barrier, which is a contextual factor) and assisting in addressing them (eg, arranging for transportation vouchers, which is a contextualized care plan). The magnitude of the effect is substantial. Converted to a number needed to treat to improve 1 red flag, contextualizing care has a number needed to treat of approximately 6 (adjusting for site).^14^ Although contextualizing care is less efficacious than, for instance, adherence therapy, which consists of 7 home visits to increase patient medication adherence (number needed to treat, 2.2), it is also much less intrusive and costly.^14^

Giving clinicians ongoing feedback on their attention to the life challenges that their patients face may be an effective strategy for heightening their awareness of and attention to social determinants of health, which may greatly improve health care outcomes and reduce health care costs. The intervention’s success in changing clinician behavior is consistent with other studies indicating that audit and feedback is an effective strategy for influencing professional practice.^15^ From the clinician’s perspective, it is an opportunity to see one’s blind spots and adjust accordingly.

### Limitations

A limitation of this study was our inability to randomize 2 of the 6 sites into the stepped wedge because of their previous exposure to the QI program. As a result, 1 site only contributed to the enhanced feedback group, and the other, though contributing to all groups, was fixed in its position in the wedge. We controlled for site in all analyses to attempt to mitigate this limitation. A limitation of the budget analysis is that estimated cost savings are based on the panel population and phase of participation in the program, rather than on individual patient contextualized care results. Our data suggest that clinicians reduce hospitalization rates by improving care to their panel, but it is possible that other confounding influences were present.

## Conclusions

Engaging patients to record their visits, when they are comfortable doing so, enables the collection of otherwise inaccessible data about attention to life context that may improve health care at a low cost. Our findings suggest that QI programs could be well advised to consider routine incorporation of training in contextualizing care through audit and feedback.
